# Genome Sequence of Selenium-Solubilizing Bacterium *Caulobacter vibrioides* T5M6

**DOI:** 10.1128/genomeA.01721-15

**Published:** 2016-02-11

**Authors:** Yihua Wang, Yanan Qin, Witold Kot, Fujun Zhang, Shixue Zheng, Gejiao Wang, Lars H. Hansen, Christopher Rensing

**Affiliations:** a Department of Plant and Environmental Science, University of Copenhagen, Frederiksberg, Denmark; b Department of Environmental Science, Aarhus University, Roskilde, Denmark; c State Key Laboratory of Agricultural Microbiology, College of Life Science and Technology, Huazhong Agricultural University, Wuhan, China; d Institute of Urban Environment, Chinese Academy of Sciences, Xiamen, China

## Abstract

*Caulobacter vibrioides* T5M6 is a Gram-negative strain that strongly solubilizes selenium (Se) mineral into Se(IV) and was isolated from a selenium mining area in Enshi, southwest China. This strain produces the phytohormone IAA and promotes plant growth. Here we present the genome of this strain containing a large number of genes encoding resistances to copper and antibiotics.

## GENOME ANNOUNCEMENT

A number of different aerobic microbes were shown to be involved in selenium (Se) oxidization under aerobic condition (1, 2). However, the whole genomes of these bacteria were not sequenced and the genes that are involved in aerobic oxidation and solubilization of selenium are not known .

*Caulobacter vibrioides* T5M6 was isolated from rhizosphere soil samples (soil Se of >5 mg/kg) in an Se mine area in Enshi, southwest China. Strain T5M6 grew better in 1/5 Luria broth (LB) and 1/10 LB than in LB at 28°C with shaking at 180 rpm. The pH of the broth varied from 7 at the beginning of growth and reached pH 8 in stationary phase. In addition, T5M6 transformed the insoluble chemical Se(0) and selenium mineral (containing Se[VI], Se[IV] Se[0], and Se[−II]) into soluble Se(IV). The 16S rRNA sequences of T5M6 revealed that it belongs to the species *Caulobacter vibrioides*, with sequence similarities of 99.04% using the EzTaxon server (3). Chromosomal DNA was purified from isolates by using a Gentra Puregen yeast/bacteria kit (Qiagen). The draft genome sequence of *C. vibrioides* T5M6 was determined by the Illumina MiSeq 2x250PE platform to generate a paired-end library. *De novo* assembly was performed with SPAdes3.5.0, resulting in 161 contigs (>200 bp). A total of 4,822 open reading frames were predicted by the RAST server and annotated using the information from GenBank and RAST.

The size of the draft genome sequence is 5,326,313 bp with an average G+C % content of 69.39. The longest assembled contig is 323,708 bp and *N*_50_ of the assembly is 71,759 bp.

We did nor measure the presence of other metals in the mine. It could contain Cr based on other similar soils that we have determined. A gene encoding a putative chromate resistance determinant was detected (*chrA*). There are a number of genes encoding metal resistance determinants including copper resistance (CopA, CopB, a P-type ATPase), and/or zinc and cadmium resistance (CzcCBA resistance-nodulation-cell division [RND]-type). Interestingly, there are no predicted resistances to arsenic and mercury. In addition, there are many potential antibiotic resistance genes such as those encoding β-lactamases.

### Nucleotide sequence accession numbers.

This whole-genome shotgun project has been deposited at DDBJ/EMBL/GenBank under the accession no. LNIY00000000. The version described in this paper is version LNIY01000000.
